# Spatial and Temporal Variation of Cultivable Communities of Co-occurring Endophytes and Pathogens in Wheat

**DOI:** 10.3389/fmicb.2016.00403

**Published:** 2016-03-31

**Authors:** Morgane Comby, Sandrine Lacoste, Fabienne Baillieul, Camille Profizi, Joëlle Dupont

**Affiliations:** ^1^Institut de Systématique, Evolution et Biodiversité—UMR 7205—Centre National de la Recherche Scientifique, MNHN, UPMC, EPHE, Muséum National D'histoire Naturelle, Sorbonne UniversitésParis, France; ^2^UFR Sciences Exactes et Naturelles—Laboratoire de Stress Défenses et Reproduction des Plantes, Moulin de la HousseReims, France; ^3^Soufflet BiotechnologiesNogent-sur-Seine, France

**Keywords:** fungi, bacteria, *Triticum aestivum*, diversity, microbial communities, biological control agents

## Abstract

The aim of this work was to investigate the diversity of endogenous microbes from wheat (*Triticum aestivum*) and to study the structure of its microbial communities, with the ultimate goal to provide candidate strains for future evaluation as potential biological control agents against wheat diseases. We sampled plants from two wheat cultivars, Apache and Caphorn, showing different levels of susceptibility to *Fusarium* head blight, a major disease of wheat, and tested for variation in microbial diversity and assemblages depending on the host cultivar, host organ (aerial organs vs. roots) or host maturity. Fungi and bacteria were isolated using a culture dependent method. Isolates were identified using ribosomal DNA sequencing and we used diversity analysis to study the community composition of microorganisms over space and time. Results indicate great species diversity in wheat, with endophytes and pathogens co-occurring inside plant tissues. Significant differences in microbial communities were observed according to host maturity and host organs but we did not find clear differences between host cultivars. Some species isolated have not yet been reported as wheat endophytes and among all species recovered some might be good candidates as biological control agents, given their known effects toward plant pathogens.

## Introduction

Plants live in close association with a diversity of bacteria and fungi, localized within or outside plants tissues. Plants-associated microorganisms are known to be beneficial, neutral, or pathogenic. Some of these symbioses are well-known, i.e., mycorrhizae (Bonfante and Anca, 2009) or plant pathogens (Gladieux et al., 2011), but others are still poorly understood, particularly endophytes. Endophytes are microorganisms, fungi or bacteria, living inside plant tissues at least part of their life cycle without causing any symptoms of disease to their host (Petrini, 1991; Wilson, 1995). Very few are obligate symbionts, which are transmitted in seeds, like the grass fungal endophyte *Epichloë* (anamorph: *Neotyphodium*). Most are facultative endophytes, which are free living generalists able to colonize plant tissues through stomata, wounds or cracks, when an opportunity arises (Hardoim et al., 2008; Rodriguez et al., 2009). Most fungal endophytes are known to be commensals or weak parasites but some appear to provide benefits to their hosts, through better responses to abiotic stresses (Rodriguez and Redman, 2008) or better defenses against pathogens (Compant et al., 2005; Gao et al., 2010) and herbivores (Clay and Schardl, 2002). However, potential fungal pathogens are also commonly isolated as endophytes and several researches have evidenced that many species that are pathogenic for some hosts may be asymptomatic for others (Malcolm et al., 2013). In addition, many fungal endophytes may switch between pathogenic and commensal or mutualistic lifestyles, depending on environmental conditions and on the host (Schulz and Boyle, 2005; Sieber, 2007; Malcolm et al., 2013). Bacterial endophytes may also have beneficial effects in inducing physiological changes that modulate the growth and development of the plant (Hardoim et al., 2008).

Non-pathogenic endophytic microorganisms might be of particular interest in the search of plant growth promoters or biological control agents (Alabouvette et al., 2006; Berg, 2009) because they are well adapted to their host, they colonize an ecological niche similar to that of phytopathogens (Berg et al., 2005) and they are often considered as good producers of secondary metabolites (Strobel and Daisy, 2003; Brader et al., 2014) required for their survival in the face of host defense responses.

Endophytes have been isolated from almost all lineages of plants and may represent a large component of microbial biodiversity (Porras-Alfaro and Bayman, 2011; Malfanova et al., 2013). But the factors driving the establishment and assemblage of species within microbial communities are still not well understood. Some studies have shown that endophyte colonization can be influenced by host species, and sometimes by different genotypes of the same species, by geographic locality, by seasonality, by different organs of the same plant and even by differences that exist within the same organ (Porras-Alfaro and Bayman, 2011).

Among the ten scientifically and/or economically most important fungal pathogens (Dean et al., 2012), four are wheat pathogens: *Puccinia* spp. causing different types of rusts, *Fusarium graminearum* one of the pathogens responsible for *Fusarium* head blight (FHB)*, Blumeria graminis* the agent of powdery mildew, and *Mycosphaerella graminicola* responsible for *Septoria tritici* blotch. All of these diseases are responsible for crop failure and yield reduction, and some, e.g., *Fusarium* species, can produce mycotoxins that are highly toxic to plants and animals, including humans (Bottalico and Perrone, 2002). No fully resistant wheat cultivar exists, so control of those diseases is primarily based on the use of fungicides along with cultural control methods to reduce the inoculum. In the present context of reduction of pesticides use and in view of the limited efficacy of fungicides against wheat diseases (Jorgensen, 2008), biological control is a promising additional control method but one that requires finding new effective microorganisms as biological control agents. Previous studies on wheat endophytes have mainly focused either on bacterial species, especially on Actinobacteria, (Zinniel et al., 2002; Coombs and Franco, 2003; Conn and Franco, 2004; Coombs et al., 2004) or on fungal species (Sieber et al., 1988; Crous et al., 1995; Vujanovic et al., 2012) and the only studies conducted on both types of microorganisms have focused on endophytes isolated from aerial organs (Larran et al., 2002, 2007) or from roots and rhizosphere (Lenc et al., 2015). In the present work, the investigation of cultivable wheat microorganisms was more diverse than in previous studies in order to maximize the diversity of microorganisms recovered, that could be further developed as biological control agents against wheat diseases.

In order to better characterize microbial communities of wheat and isolate strains with potential beneficial applications, the main objectives of the present study were to:

- examine the abundance and diversity of cultivable microorganisms, fungi, and bacteria, living inside wheat plants tissues.
- study the effects of host genotype, host organs and host maturity on the distribution of cultivable microorganisms in wheat plants.

## Materials and methods

### Sampling

Field samplings were conducted between May and July 2012 at Cucharmoy, France, (48° 35′ 00″ N 3° 11′ 40″ E) on untreated experimental plots of Soufflet Agriculture. The preceding crop was peas (*Pisum sativum*) in 2011 and wheat (*Triticum aestivum*) in 2010. Plants from two wheat cultivars Caphorn and Apache, known to differ in their susceptibility to FHB, were collected at heading (Growth Stage 59, according to the code defined by Zadoks et al., 1974), flowering (GS 61-69) and mealy ripe (GS 77-79). Apache is more resistant than Caphorn to FHB, with resistance levels near seven and three, respectively on the resistance scale to FHB going from 2 (most susceptible cultivar) to 7 (most resistant cultivar; Arvalis communication). Development stages were chosen as to cover the critical period for infection by FHB, when conidia and ascospores of pathogens could infect the heads. Plots from both cultivars were separated only by a few meters and, therefore, benefitted from the same soil and growth conditions. Four plants from both cultivars were sampled at each stage of development, with Apache developing 2 to 3 days in advance of Caphorn. Due to favorable climatic conditions, fungal diseases could be observed in the experimental plots and sampling plants were chosen that displayed the fewest symptoms of diseases with the hypothesis that endophytes may have protected the plants against the growth or activity of pathogens.

### Isolation of endogenous microbes

Roots and aerials organs, including leaves, stems, anthers, glumes, rachis, and kernels, were surface-sterilized by dipping them in 70% EtOH for 2 min, in 0.5% NaOCl for 2 min, in EtOH 70% for 1 min, followed by a brief rinse in sterile distilled water. Five fragments per organ, 25 mm long, were taken from each plant, except for anthers, rachis, and kernels where organs were taken entirely. Then each fragment (or organ) was cut into five pieces inoculated together in a Petri plate containing malt-agar medium (MA). The culture medium selected for the isolation of microorganisms, MA, was chosen as to favor fungi and bacteria able to grow in the same conditions as wheat pathogens, with the idea that they will have a better chance to compete with and control them. Five plates per organ were obtained for each of the 24 plants sampled. In total, we sampled 600 roots fragments and 1816 aerial plant organs fragments. The surface sterilization of the plant material was checked by rolling the sterilized plant material onto MA. Plates were incubated at 25°C with ambient light and checked daily for fungal and bacterial growth up to 2 weeks, until plates' invasion. Emergent colonies were picked and transferred on MA (for fungi) or LB (Lysogenic Broth, for bacteria) for isolation into pure cultures. Monospores isolations using dilution method were made to purify fungal isolates. Bacteria were purified by streaking, in order to isolate one single colony.

### Sequencing and molecular identification

For fungal isolates, genomic DNA was extracted from fresh mycelium grown on MA. Extractions were performed using the DNeasy Plant Mini Kit (Qiagen, Ltd., Crawley, UK) following the manufacturer's instructions. ITS plus the 5′ end of 28S rDNA were amplified using primers sets ITS4/ITS5 (White et al., 1990) and LROR/LR6 (Vilgalys and Hester, 1990; Vilgalys and Sun, 1994) respectively. PCR amplifications were performed using a BioRad DNA Engine Peltier Thermal cycler with 30 cycles of 30 s at 94°C, 30 s at 55°C (for ITS4/ITS5 primers), or 50°C (for LROR/LR6 primers), 40 s at 72°C; 10 min at 72°C, in a 25 μL reaction mix, containing 12.5 μL genomic DNA (dilution: 10^−2^ after extraction), 5 μL PCR Direct Loading Buffer with MgCl_2_ (Q-Biogen), 0.5 μL dNTPs (6.25 mM, dNTP Mix, Q-Biogen), 1 μL of each 10 μM primer (Eurogentec), 0.125 μL Taq DNA Polymerase (Q-Biogen, 5 units/μL), and 4.875 μL sterile water. For bacterial isolates, the 3′ end of 16S rDNA was directly amplified from one colony diluted in 1 mL sterile water using primers set 27F/1492R (Wilson et al., 1990). PCR amplifications were performed using a BioRad DNA Engine Peltier Thermal cycler with 5 min at 94°C; 30 cycles of 60 s at 94°C, 60 s at 53°C, 2 min at 72°C; 10 min at 72°C, in 50 μL reaction mix, containing 4 μL of bacterial suspension, 10 μL of Green Flexi Buffer (x5, Promega), 3 μL MgCl_2_ (25 mM, Promega), 0.2 μL dNTPs (25 mM, Q-Biogen), 5 μL of each 2 μM primer (Eurogentec), 0.26 μL Taq DNA Polymerase (Go Taq Promega, 5 units/μL), and 22.34 μL sterile water. PCR products were purified and sequenced by Genoscreen (Lille, France) in both directions to confirm the accuracy of each sequence. Sequences were assembled with CodonCode Aligner v. 3.7.1 (Codon Code Corporation), checked by visual inspection of the chromatograms and edited if necessary. Sequences were identified using the BLAST option at http://blast.st-va.ncbi.nlm.nih.gov/Blast.cgi. Best hits were carefully examined to attribute species names (≥97% of sequence similarities).

### Culture collection and nucleotide sequence accession numbers

All microbial isolates have been deposited at Soufflet Biotechnologies. Sequences have been deposited in the GenBank database under accession numbers from KT692544-KT692597, KT699061-KT699075.

### Analysis of data

Diversity was measured using Shannon and Pielou indices. In addition to the total dataset a second dataset was created excluding singletons, that is, species that were isolated only once during the study, and this dataset was used for analysis of endophyte assemblages. Species accumulation curves were computed for both datasets in EstimateS v.9.1.0. (Colwell et al., 2012) (http://viceroy.eeb.uconn.edu/EstimateS) and compared with ACE and Chao1 curves. Variability in the endophyte assemblages in the two cultivars, at different stages of development and in different parts of plants (roots vs. aerial organs) was measured using permutational analysis of variance (PERMANOVA, 9999 permutations, use of Bray-Curtis dissimilarities) with the package vegan (Oksanen et al., 2013) and correspondence analysis with the package ade4 (Thioulouse et al., 1997) in R software (http://www.r-project.org/). We performed an indicator species test (Dufrene and Legendre, 1997) using the package labdsv (Roberts, 2010) in R software to identify species characteristic of each stage of development and each type of organs. Indicator species are defined as the most characteristic species of each group, that is, those species most nearly confined to the group and present in most samples belonging to that group.

## Results

### Global diversity and phylogenetic relationships of plants microbiotes

**Figure 1:**
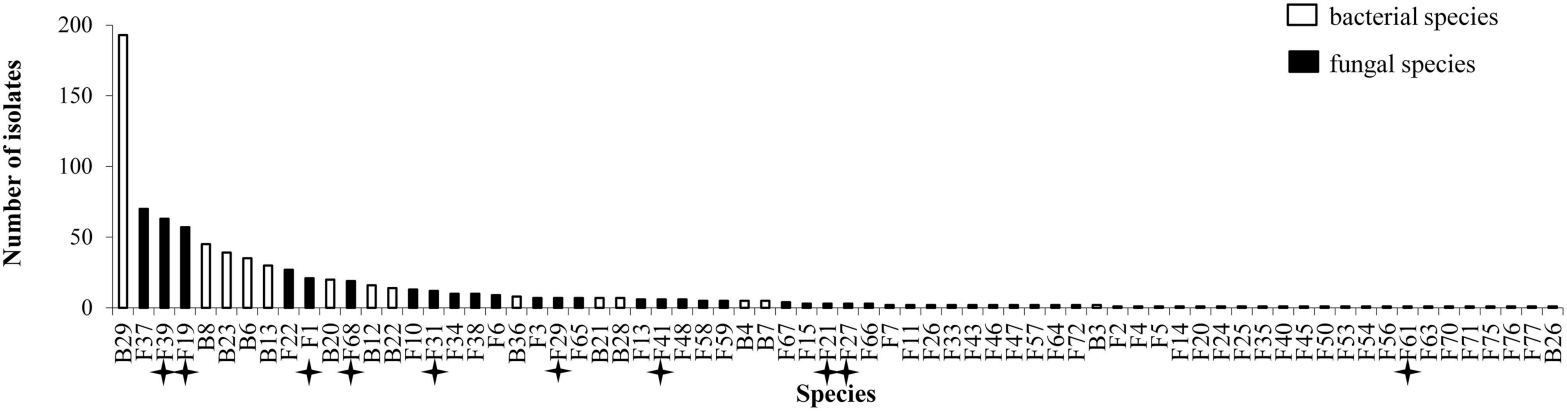
**Abundance of isolates for 70 species isolated from inner tissues of 24 wheat plants**. Stars indicate known pathogens of wheat.

All plants sampled harbored fungi and bacteria in their inner tissues. Overall 55 fungal species (416 isolates) and 15 bacterial species (427 isolates) have been isolated in this study (Table 1). Fungi were more abundant in Apache (244 isolates) and bacteria were more abundant in Caphorn (250 isolates). Species richness was high in all plants and quite similar in both wheat cultivars, with Shannon indices ranging from 2.12 to 3.68 in Caphorn and from 2.45 to 3.90 in Apache (S1 Table). The richness recovered at flowering in both wheat cultivars was lower (with Shannon indices ranging from 2.12 to 2.91 in Caphorn and from 2.45 to 2.97 in Apache) than at the earlier and later stages of heading or mealy ripe respectively. Species appeared relatively well distributed in plants, with Pielou indices near 1 in most plants, especially at heading and mealy ripe (S1 Table), indicating only few dominant species. Pielou indices were lower at flowering (0.67–0.84 in Caphorn and 0.77–0.89 in Apache) than heading or mealy ripe (S1 Table) indicating more dominant species at that stage of plant development which was in accordance with lower species richness. Globally, there were few dominant species (≥50 isolates over the whole study), namely *Pseudomonas trivialis* (B29), *Didymella exitialis* (F19), *Alternaria infectoria* (F37), and *Microdochium nivale* (F39) and many rare or singletons species (37 species with ≤ 3 isolates, of which 22 singletons; Figure 1). Depending on plants, singletons represented 0 to 15.8% of the total number of species isolated (S1 Table). When singletons were removed from the analysis, species accumulation curves reached an asymptote and met ACE and Chao1 curves (S1 Figure), indicating that our sampling recovered all common species. However, when including singletons, accumulation curves did not reach the asymptote (S1 Figure), indicating that more rare species would be isolated with additional sampling.

**Table 1:** **Species of fungi (F) and bacteria (B) isolated from inner tissues of the two wheat cultivars Caphorn and Apache**.

**Species**	**Codes**	Phylum^**^/Class	**Order**	**Number of isolates from:**		**Pathogens from**
				**Caphorn**	**Apache**	
***Alternaria triticimaculans***	F1	A/Dothideomycetes	Pleosporales	10	11	Wheat (Perello and Larran, 2013)
^*^Athelia bombacina	F2	B/Agaricomycetes	Atheliales	0	1	
*Aureobasidium proteae*	F3	A/Dothideomycetes	Dothideales	5	2	
^*^Clonostachys rosea	F4	A/Sordariomycetes	Hypocreales	0	1	
*Biscogniauxia nummularia	F5	A/Sordariomycetes	Xylariales	1	0	
***Botrytis cinerea***	F6	A/Leotiomycetes	Helotiales	1	8	Broad host range (Dean et al., 2012)
*Chaetomium globosum*	F7	A/Sordariomycetes	Sordariales	2	0	
*Cladosporium allii*	F10	A/Dothideomycetes	Capnodiales	7	6	
*Cladosporium halotolerans*	F11	A/Dothideomycetes	Capnodiales	2	0	
*Coriolopsis gallica*	F13	B/Agaricomycetes	Polyporales	0	6	
^*^Cytospora chrysosperma	F14	A/Sordariomycetes	Diaportales	0	1	
*Diaporthe eres*	F15	A/Sordariomycetes	Diaportales	0	3	
***Didymella exitialis***	F19	A/Dothideomycetes	Pleosporales	18	39	Wheat (Punithalingam, 1979)
^*^Doratomyces microsporus	F20	A/Sordariomycetes	Microascales	0	1	
***Drechslera poae***	F21	A/Dothideomycetes	Pleosporales	0	3	Wheat (Wiese, 1987)
*Epicoccum nigrum*	F22	A/Dothideomycetes	Pleosporales	7	20	
^*^Eutypa maura	F24	A/Sordariomycetes	Xylariales	1	0	
^*^Funalia trogii	F25	B/Agaricomycetes	Polyporales	0	1	
***Fusarium redolens***	F26	A/Sordariomycetes	Hypocreales	1	1	Peas/Wheat (Taheri et al., 2011)
***Fusarium tricinctum***	F27	A/Sordariomycetes	Hypocreales	0	3	Wheat (Xu and Nicholson, 2009)
***Gaeumannomyces graminis***	F29	A/Sordariomycetes	-	4	3	Wheat ((Freeman and Ward, 2004)
***Fusarium graminearum***	F31	A/Sordariomycetes	Hypocreales	10	2	Wheat (Xu and Nicholson, 2009; Dean et al., 2012)
*Gnomoniopsis idaeicola*	F33	A/Sordariomycetes	Diaportales	2	0	
*Hyphodermella rosae*	F34	B/Agaricomycetes	Polyporales	0	10	
^*^Ilyonectria macrodidyma	F35	A/Sordariomycetes	Hypocreales	1	0	
***Alternaria infectoria***	F37	A/Dothideomycetes	Pleosporales	29	41	Peas (Perello and Larran, 2013)
*Microdochium bolleyi*	F38	A/Sordariomycetes	Xylariales	6	4	
***Microdochium nivale***	F39	A/Sordariomycetes	Xylariales	24	39	Wheat (Xu and Nicholson, 2009)
^*^Mortierella alpina	F40	Z/-	Mortierellales	1	0	
***Mycosphaerella graminicola***	F41	A/Dothideomycetes	Capnodiales	4	2	Wheat (Dean et al., 2012; Miedaner et al., 2013)
*Dichotomomyces cejpii*	F43	A/Eurotiomycetes	Eurotiales	1	1	
^*^Ophiosphaerella sp.	F45	Dothideomycetes	Pleosporales	0	1	
*Oxyporus latemarginatus*	F46	B/Agaricomycetes	Hymenochaetales	0	2	
*Peniophora cinerea*	F47	B/Agaricomycetes	Russulales	0	2	
*Periconia macrospinosa*	F48	A/Dothideomycetes	Pleosporales	3	3	
^*^Parastagonospora avenae	F50	A/Dothideomycetes	Pleosporales	0	1	
^*^Phlebia subserialis	F53	B/Agaricomycetes	Polyporales	0	1	
^*^Phoma caloplacae	F54	A/Dothideomycetes	Pleosporales	1	0	
^*^Podospora fimbriata	F56	A/Sordariomycetes	Sordariales	0	1	
*Podospora glutinans*	F57	A/Sordariomycetes	Sordariales	0	2	
*Polyporus lepideus*	F58	B/Agaricomycetes	Polyporales	4	1	
*Peniophora* sp.	F59	B/Agaricomycetes	Russulales	0	5	
^*^Pyrenophora tritici-repentis	F61	A/Dothideomycetes	Pleosporales	1	0	Wheat (Ciuffetti and Tuori, 1999)
^*^Rhodosporidium kratochvilovae	F63	B/Exobasidiomycetes	Sporidiales	1	0	
*Sarocladium kiliense*	F64	A/Sordariomycetes	Hypocreales	2	0	
***Sclerotinia sclerotiorum***	F65	A/Leotiomycetes	Helotiales	7	0	Broad host range (Bolton et al., 2006)
*Stereum hirsutum*	F66	B/Agaricomycetes	Russulales	1	2	
*Talaromyces flavus*	F67	A/Eurotiomycetes	Eurotiales	3	1	
***Rhizoctonia solani***	F68	B/Agaricomycetes	Cantharellales	10	9	Broad host range (Dean et al., 2012)
*Trametes gibbosa	F70	B/Agaricomycetes	Polyporales	0	1	
*Trametes hirsuta	F71	B/Agaricomycetes	Polyporales	1	0	
*Trametes versicolor*	F72	B/Agaricomycetes	Polyporales	1	1	
*Xylaria longipes	F75	A/Sordariomycetes	Xylariales	1	0	
*Cladosporium iridis	F76	A/Dothideomycetes	Capnodiales	0	1	
*Ganoderma carnosum	F77	B/Agaricomycetes	Polyporales	0	1	
*Bacillus amyloliquefaciens*	B3	F/Bacilli	Bacillales	2	0	
*Bacillus cereus*	B4	F/Bacilli	Bacillales	5	0	
*Bacillus megaterium*	B6	F/Bacilli	Bacillales	21	14	
*Bacillus pumilus*	B7	F/Bacilli	Bacillales	5	0	
*Bacillus subtilis*	B8	F/Bacilli	Bacillales	12	33	
***Erwinia aphidicola***	B12	P/Gammaproteobacteria	Enterobacteriales	10	6	Peas (Santos et al., 2009)
***Erwinia persicina***	B13	P/Gammaproteobacteria	Enterobacteriales	29	1	Peas (Zhang and Nan, 2014)
*Paenibacillus hordei*	B20	F/Bacilli	Bacillales	11	9	
*Paenibacillus peoriae*	B21	F/Bacilli	Bacillales	7	0	
*Pantoea agglomerans*	B22	P/Gammaproteobacteria	Enterobacteriales	14	0	
*Pantoea vagans*	B23	P/Gammaproteobacteria	Enterobacteriales	27	12	
^*^Pseudomonas fluorescens	B26	P/Gammaproteobacteria	Pseudomonadales	0	1	
*Pseudomonas lurida*	B28	P/Gammaproteobacteria	Pseudomonadales	7	0	
*Pseudomonas trivialis*	B29	P/Gammaproteobacteria	Pseudomonadales	100	93	
*Stenotrophomonas africana*	B36	P/Gammaproteobacteria	Xanthomonadales	0	8	

*Data about pathogenicity are reported from the literature (references in brackets)*.

Species in bold are known pathogens of wheat and/or peas, the preceding crop.

** A, Ascomycota; B, Basidiomycota; Z, Zygomycota; F, Firmicutes; P, Proteobacteria.

* Singletons are identified by.

Species isolated were phylogenetically very diverse (Table 1). Fungal species belonged to Ascomycota (75.7%), Basidiomycota (22.9%), and Zygomycota (1.43%). Most Ascomycota were Sordariomycetes (35.8%) or Dothideomycetes (26.4%) and most Basidiomycota were Agaricomycetes (93.8%) in which Polyporales and Russulales were two of the most abundant orders. Bacterial species belonged to the class Gammaproteobacteia of the Proteobacteria (53.3%) and the class Bacilli of the Firmicutes (46.7%).

### Distribution patterns of plants microbiotes

To compare the species assemblages of microbial communities, the data obtained from the four plants collected for each cultivar at each stage of development were pooled.

#### Between the two host cultivars

**Figure 2:**
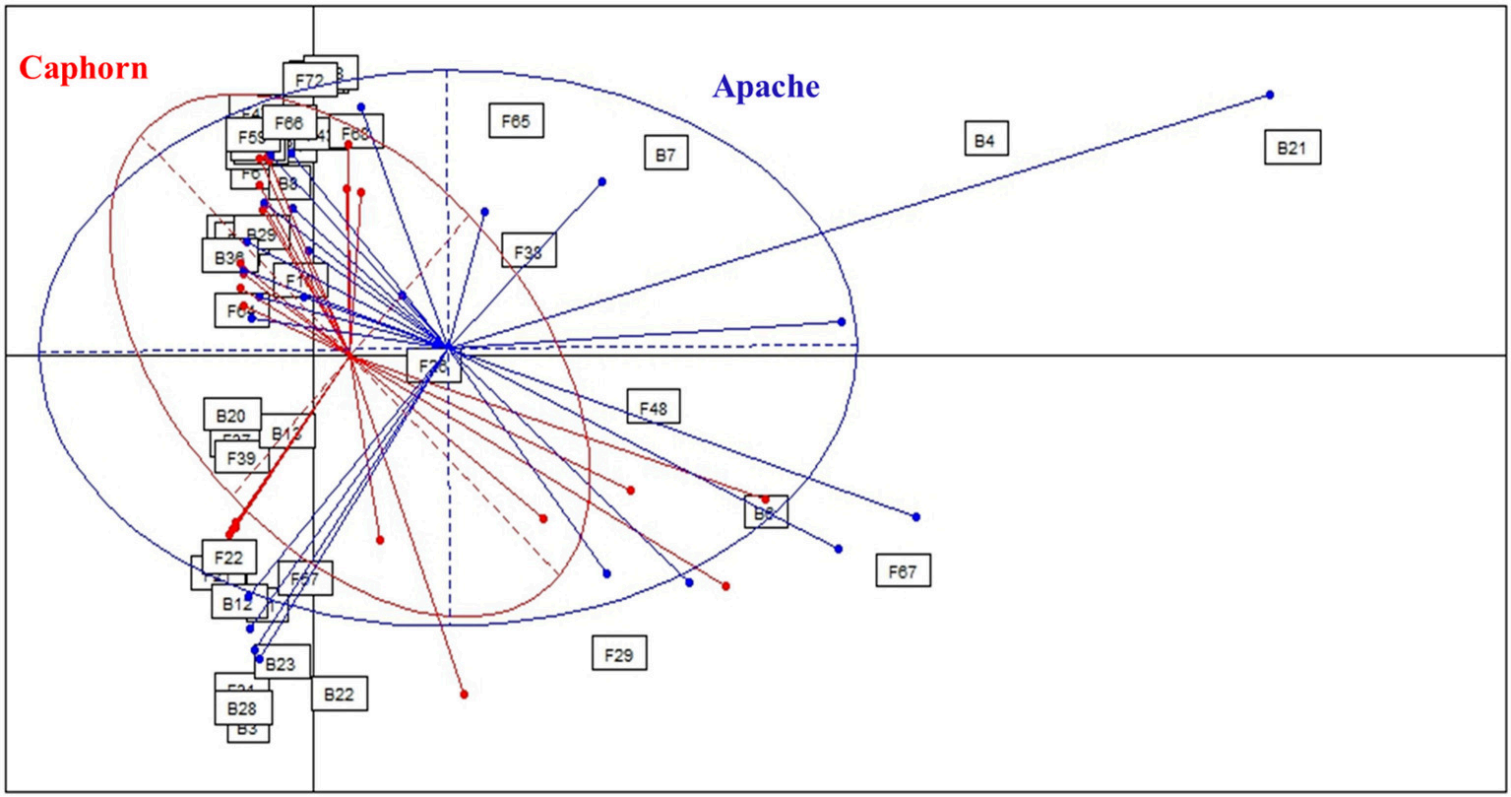
**Comparison of microbial assemblages in wheat plants depending on the host cultivar, using correspondence analysis**. Based on non-singleton taxa. Components 1 and 2 of the correspondence analysis explained respectively 12.9 and 11.3% of the total inertia.

PERMANOVA analysis showed a significant effect of the cultivar on microorganism assemblages inside the plants (*p*-value = 0.0020). However, the effect of that factor was weak (*F* = 3.24). Correspondence analysis, based on non-singleton species, evidenced an overlap between microbial communities from each cultivar (Figure 2). The two cultivars shared 20 fungal species and seven bacterial species (Table 1), including the most abundant taxa (B29, F37, F39, F19), representing 83 and 88.5% of fungal and bacterial isolates, respectively. Each cultivar harbored unique taxa but those were rare, particularly for fungi. From a total of 55 fungi and 15 bacteria found in our study, Caphorn had 14 unique fungal species (13 with three or fewer isolates, of which nine were singletons) and six unique bacterial species and Apache had 21 unique fungal species (18 with three or fewer isolates, of which 12 were singletons) and two unique bacterial species (Table 1).

Given that microbial communities were not strongly different depending on the cultivar, we pooled the species across cultivars to form a single community for subsequent analysis.

#### Between aerial organs and roots

**Figure 3:**
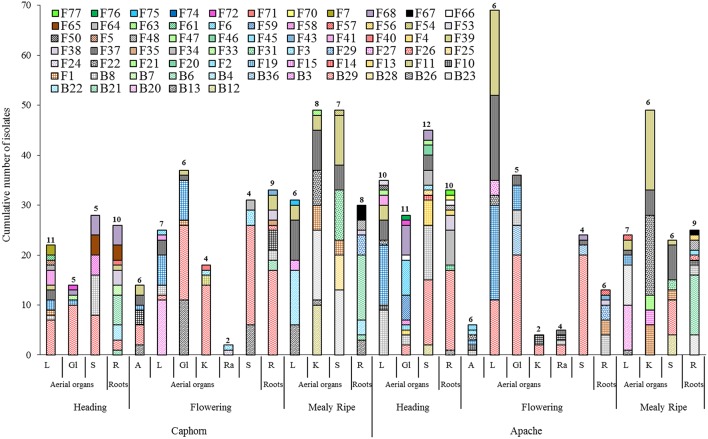
**Species richness within aerial organs (L, leaves; Gl, Glumes; S, stems; A, anthers; K, kernels; Ra, rachis) and roots (R) from two wheat cultivars (Caphorn and Apache) at heading, flowering, and mealy ripe stages of development**. Numbers indicated above bars correspond to the number of species recovered from the sample considered.

Every organ provided microorganisms but the communities appeared very diverse taxonomically and in terms of the number and abundance of species (Figure 3). Samples contained between two and 12 different species, with variable numbers of singletons (half or more species were singletons in 12 of the 30 samples so considered). It was not possible to analyse the proportion of fungal vs. bacterial species because of the experimentation biases favoring fungi. Among the 30 samples, the highest numbers of species were obtained from the roots, leaves, and stems.

**Figure 4:**
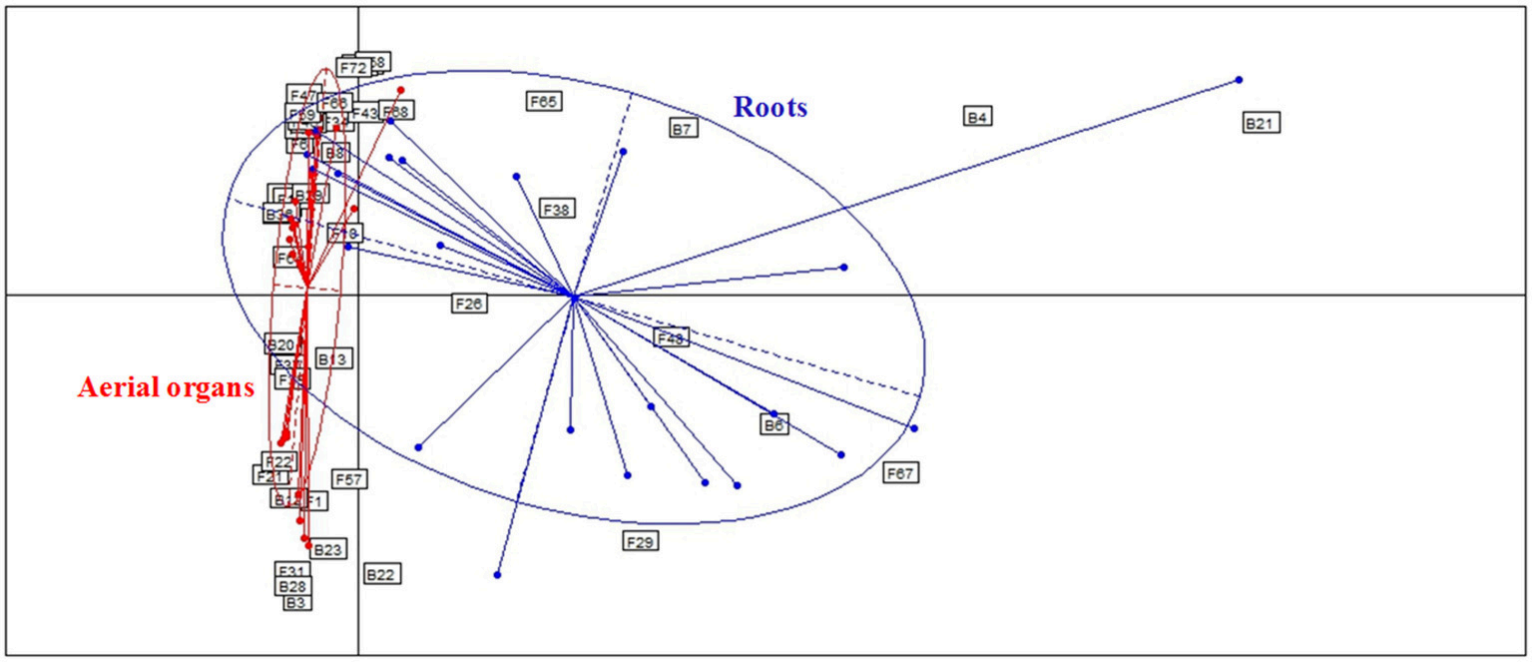
**Comparison of microbial assemblages in wheat plants either in roots or aerial organs, using correspondence analysis**. Based on non-singleton taxa. Components 1 and 2 of the correspondence analysis explained respectively, 12.9 and 11.3% of the total inertia.

Globally, when aerial organs were compared to the roots, microbial communities were found to be significantly different using PERMANOVA analysis (*p*-value = 0.0001, *F* = 23.6) and correspondence analysis (Figure 4). The indicator species analysis identified three species characteristic of roots that were completely absent from aerial organs (indicator values ranging from 0.17 to 0.48) and 14 indicator species associated with aerial parts of plants (indicator values ranging from 0.21 to 0.83), of which seven were unique to aerial organs (Table 2). Among those, *A. infectoria* (F37) and *M. nivale* (F39) colonized extensively the shoots while others were isolated from only one organ, as *F. graminearum* (F31) from the stems and *Paenibacillus hordei* (B20) from the leaves.

**Table 2:** **Indicator species analysis determining species characteristic of each type of organs**.

**Indicator species**	**Times of appearance in:**		**Cluster**	**Indicator value**	**Probability**
	**Aerial organs**	**Roots**			
F37—*Alternaria infectoria*	70	0	Aerial organs	0.8333	0.001
F39—*Microdochium nivale*	59	4	Aerial organs	0.7004	0.001
B29—*Pseudomonas trivialis*	158	35	Aerial organs	0.6769	0.001
F19—*Didymella exitialis*	57	0	Aerial organs	0.6667	0.001
F22—*Epicoccum nigrum*	27	0	Aerial organs	0.4583	0.001
B8—*Bacillus subtilis*	37	8	Aerial organs	0.4080	0.024
F1—*Alternaria triticimaculans*	18	3	Aerial organs	0.3549	0.010
B13—*Erwinia persicina*	27	3	Aerial organs	0.2987	0.035
B23—*Pantoea vagans*	35	4	Aerial organs	0.2978	0.040
B12—*Erwinia aphidicola*	16	0	Aerial organs	0.2917	0.011
B20—*Paenibacillus hordei*	20	0	Aerial organs	0.2917	0.013
F68—*Rhizoctonia solani*	15	4	Aerial organs	0.2608	0.040
F31—*Fusarium graminearum*	12	0	Aerial organs	0.2500	0.028
F3—*Aureobasidium protae*	7	0	Aerial organs	0.2083	0.034
B6—*Bacillus megaterium*	0	35	Roots	0.4783	0.001
F38—*Microdochium bolleyi*	0	10	Roots	0.3478	0.003
F29—*Gaeumannomyces graminis*	0	7	Roots	0.1739	0.047

#### Between stages of development

**Figure 5:**
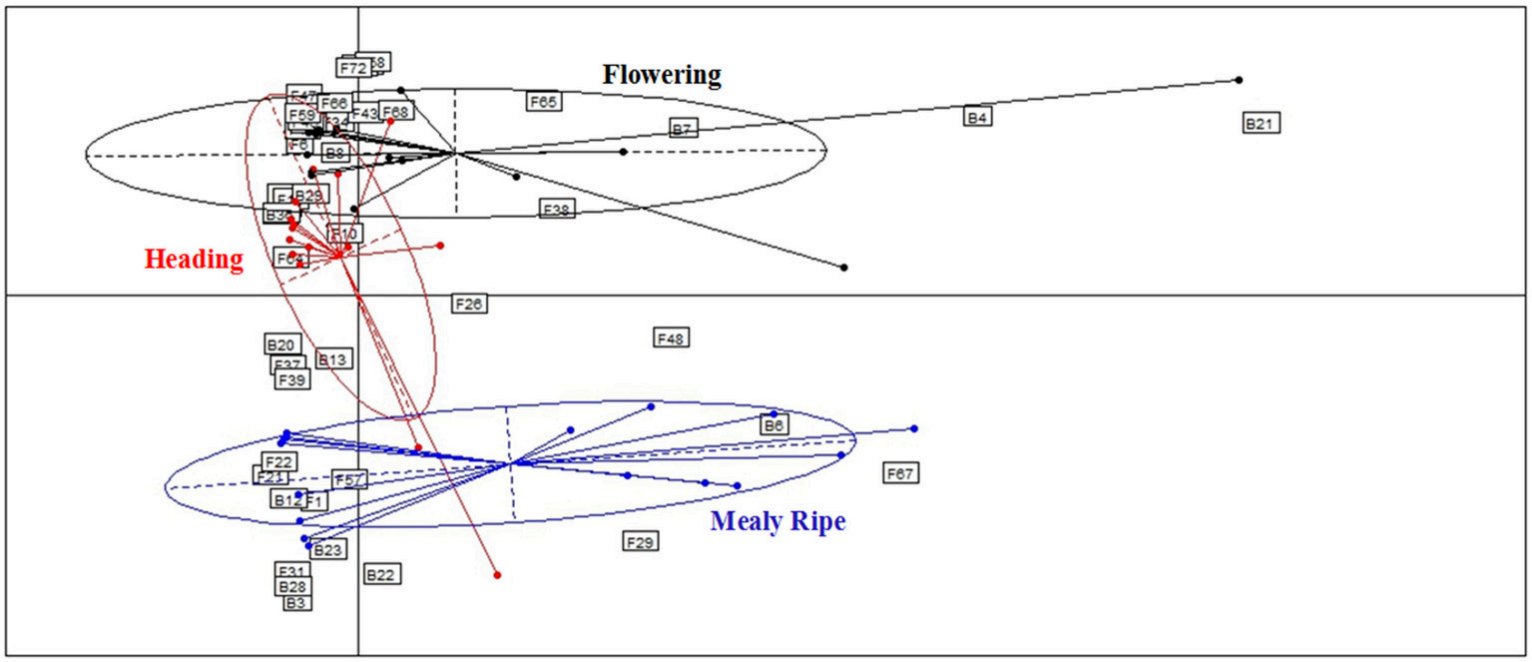
**Comparison of microbial assemblages in wheat plants depending on host maturity, using correspondence analysis**. Based on non-singleton taxa. Components 1 and 2 of the correspondence analysis explained respectively, 12.9 and 11.3% of the total inertia.

**Figure 6:**
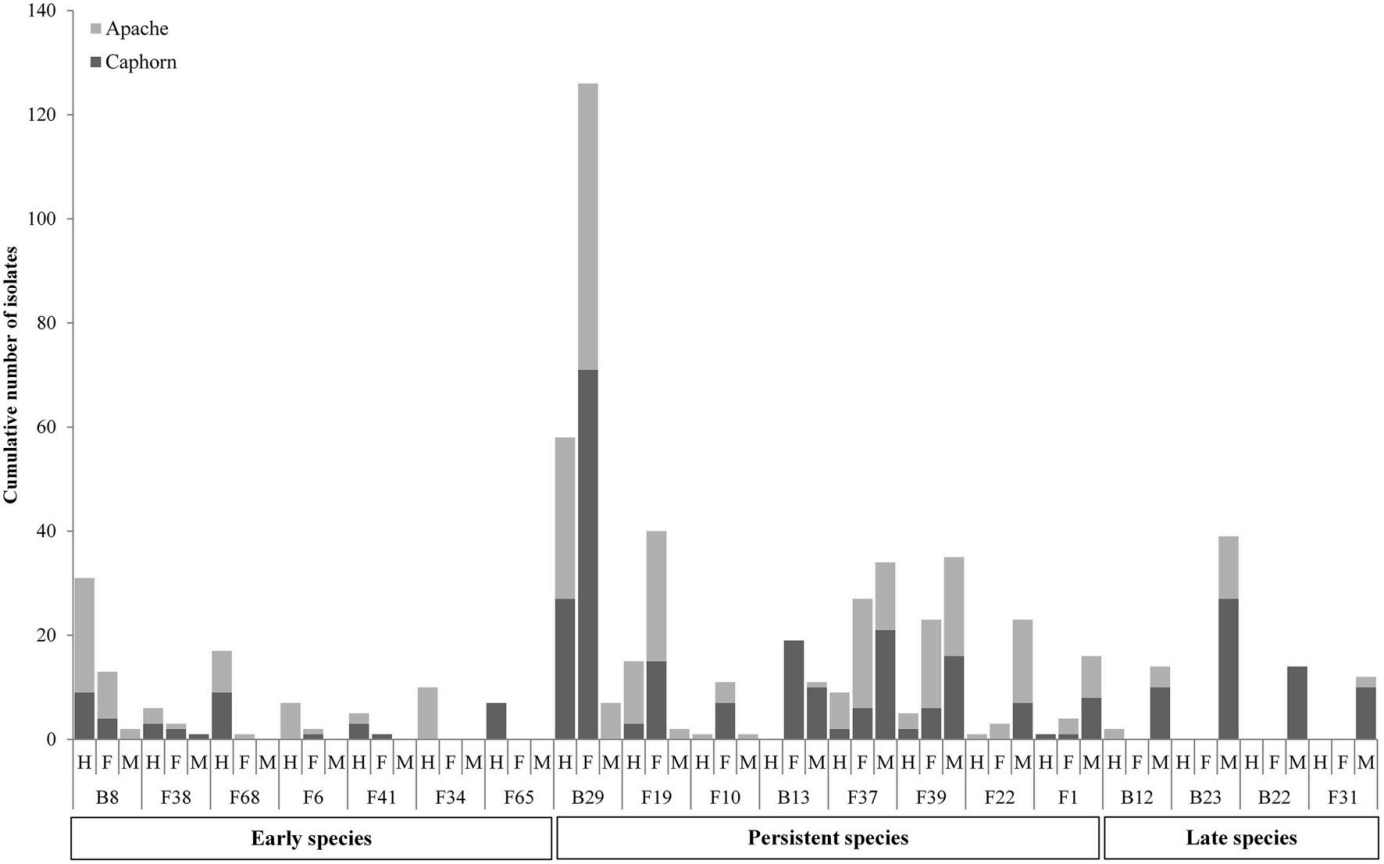
**Evolution of species during wheat development (H, Heading; F, Flowering; M, Mealy Ripe) in the two cultivars Caphorn and Apache**. Only relevant species are reported.

PERMANOVA analysis indicated a significant effect of the host maturity on microorganism assemblages (*p*-value = 0.0001, *F* = 14.9). Correspondence analysis told the same story, with species from the same stage of development clustering together in correspondence analysis (Figure 5). We observed a succession of species on plots during wheat development (Figure 6) with a group of early species, only present at heading or with a high incidence: *Bacillus subtilis* (B8)*, Microdochium bolleyi* (F38), *Rhizoctonia solani* (F68)*, Botrytis cinerea* (F6), *M. graminicola* (F41), *Sclerotinia sclerotiorum* (F65), and all the Agaricomycetes. Some early species persisted at flowering and declined at mealy ripe: *P. trivialis* (B29), *D. exitialis* (F19), *Cladosporium allii* (F10), and *Erwinia persicina* (B13). Other species increased from heading to flowering and mealy ripe: *A. infectoria* (F37), *M. nivale* (F39), *Epicoccum nigrum* (F22), *Alternaria triticimaculans* (F1), *Gaeumannomyces graminis* (F29), and *Erwinia aphidicola* (B12). There were also late species present only at mealy ripe: *Pantoea vagans* (B23), *Pantoea agglomerans* (B22), *F. graminearum* (F31), *Drechslera poae* (F21), and the yeast *Rhodosporidium kratochvilovae* (F63). The indicator species analysis identified some species characteristic of each stage of plant development: four species were associated with heading (indicator values ranging from 0.25 to 0.53), six species were associated with flowering (indicator values ranging from 0.20 to 0.53) and seven species were associated with mealy ripe (indicator values ranging from 0.33 to 0.69) (Table 3).

**Table 3:** **Indicator species analysis determining species characteristic of each stage of wheat development**.

**Indicator species**	**Times of appearance at:**			**Cluster**	**Indicator value**	**Probability**
	**Heading**	**Flowering**	**Mealy ripe**			
F68—*Rhizoctonia solani*	18	1	0	Heading	0.5310	0.001
F34—*Hyphodermella rosae*	10	0	0	Heading	0.2500	0.026
F65—*Sclerotinia sclerotiorum*	7	0	0	Heading	0.2500	0.023
B4—*Bacillus cereus*	5	0	0	Heading	0.2500	0.037
B29—*Pseudomonas trivialis*	60	126	20	Flowering	0.5339	0.005
F10—*Cladosporium allii*	1	11	2	Flowering	0.4557	0.001
F19—*Didymella exitialis*	15	40	8	Flowering	0.3337	0.032
F3—*Aureobasidium protae*	1	6	1	Flowering	0.2306	0.020
F27—*Fusarium tricinctum*	0	3	1	Flowering	0.2000	0.025
B36—*Stenotrophomonas africana*	0	8	2	Flowering	0.2000	0.023
B23—*Pantoea vagans*	0	0	39	Mealy ripe	0.6875	0.001
F22—*Epicoccum nigrum*	1	3	23	Mealy ripe	0.4228	0.003
B6—*Bacillus megaterium*	6	2	27	Mealy ripe	0.3843	0.005
F1—*Alternaria triticimaculans*	1	4	16	Mealy ripe	0.3762	0.010
F31—*Fusarium graminearum*	0	0	12	Mealy ripe	0.3750	0.003
B22—*Pantoea agglomerans*	0	0	14	Mealy ripe	0.3750	0.002
B12—*Erwinia aphidicola*	2	0	14	Mealy ripe	0.3281	0.014

## Discussion

We explored the cultivable endogenous microbiota of wheat plants from two cultivars known to differ in their susceptibility to FHB, in order to better characterize the diversity and distribution patterns of microbes that could be further exploited as biological control agents against wheat diseases such as FHB. It is important to remember that, at the time of the sampling, all plants displayed diverse visual symptoms of diseases, due to climatic conditions highly favorable to fungal infections that occurred during seedling growth. We sampled plants with the fewest symptoms, having postulated that these plants should harbor a more diverse community of endophytes potentially useful in plant protection. We deliberately chose a culture dependant approach, instead of cultivation-independent metagenomics approaches, because we needed living microorganisms to evaluate in the future their potential to control wheat pathogens.

### Global diversity of the microbial communities

Overall, great species richness was observed (Table 1) with few dominant species and many rare taxa (Figure 1), following the community structures in many ecosystems (Magurran and Henderson, 2003), and particularly in fungal endophyte communities (Arnold and Lutzoni, 2007). The significance of rare taxa remains unclear, we do not even know if they are active or simply awaiting favorable environmental conditions to become active. More fungi (55 species) than bacteria (15 species) have been isolated, which might reflect a sampling bias in favor of aerial organs, which are favored by fungi including pathogens (Xu and Nicholson, 2009; Miedaner et al., 2013; Perello and Larran, 2013), but more importantly which must result from the use of an isolation method more appropriate for fungi than for bacteria. However, given that the same sterilization procedure, growth medium, and incubation conditions have been applied to all collected samples, the comparison of microbial communities across cultivars, host organs, and development stages should be unaffected by any bias related to the isolation procedure. Over the 55 fungal species isolated in this study, only 22 were reported at least in one of the previous studies on wheat fungal endophytes (S2 Table) with *Alternaria, Acremonium, Cladosporium, Phoma* spp., *Chaetomium globosum*, and *E. nigrum* being the most frequently isolated (in four or five of the studies considered). These fungi are ubiquitous saprophytes, sporulating in soil or on dead leaf material (Hayes, 1979) and have been reported as opportunistic colonizers of many plants (Petrini, 1991). They may be part of a “core microbiome” as defined by Shade et al. (2014) concerning generalists, persistent members of microbial populations in apple flowers. The most important wheat pathogens known from the literature were isolated (15 species, see Table 1) and they were among the 10 most abundant species (Figure 1). Remarkably, an important diversity of non-wheat pathogenic fungi was also isolated, of which many species have not yet been reported as wheat endophytes, such as *Hyphodermella rosae, Coriolopsis gallica, Diaporthe eres, Gnomoniopsis idaeicola, Dichotomomyces cejpii, Peniophora* spp., *Podospora glutinans, Polyporus lepideus, Stereum hirsutum, Talaromyces flavus*, and *Trametes versicolor*, found as non-singleton in this study; however these were in the long tail of low-abundant taxa (≤ 10 isolates, Figure 1). It is of interest to note that Basidiomycota are usually isolated in small number as endophytes, and mostly isolated from trees (Rungjindamai et al., 2008; Martin et al., 2015). In agricultural soil, species such as *H. rosae*, frequently isolated in this study, together with *C. gallica, Peniophora* spp., *S. hirsutum* or *Trametes* spp., others wood-decaying fungi isolated with low abundance, are more likely to be found on lignicolous plant debris, producing at maturity numerous spores discharged in the air, contributing to the air-borne inoculum that may enter the aerial plant tissues and colonize them as endophytes. All these hyper diverse fungi recovered, mostly isolated from the shoot, may correspond to class 3 endophytes defined by Rodriguez et al. (2009), and distinguished from other endophytes classes by horizontal transmission, the formation of higly localized infections and the potential to confer benefits or costs on hosts that are not necessarily habitat-specific.

Concerning bacteria, researches have so far mainly focused on wheat endophytic actinobacteria (Coombs and Franco, 2003; Conn and Franco, 2004; Coombs et al., 2004) or on rhizospheric bacteria of wheat (McSpadden Gardener and Weller, 2001; Velázquez-Sepúlveda et al., 2012; Yin et al., 2013; Donn et al., 2015; Lenc et al., 2015). The rhizosphere is assumed to be the main source of bacterial endophytic colonizers (Bulgarelli et al., 2013; Malfanova et al., 2013), although this opinion was contradicted by results from high-throughput sequencing approaches showing that root bacterial and fungal endophytic communities of *Populus deltoides* trees were distinct assemblages rather than opportunistic subsets of the rhizosphere (Gottel et al., 2011). Among the 13 to 24 genera described from the rhizosphere of wheat by the authors previously mentioned (McSpadden Gardener and Weller, 2001; Velázquez-Sepúlveda et al., 2012; Yin et al., 2013; Donn et al., 2015; Lenc et al., 2015), only four genera were found in our study, and may have penetrated the roots from the rhizosphere: *Pseudomonas* (three species) with *P. trivialis* accounting for almost half of the total bacterial isolates (Figure 1), *Erwinia* (two species), *Pantoea* (two species), and *Bacillus* (five species). Two additional genera, *Paenibacillus* and *Stenotrophomonas* were isolated with low frequency. However, isolation methods were not identical in all these studies, making a strict comparison difficult because different culture media and incubation conditions can enhance significantly the diversity of bacterial collection (Park et al., 2013).

### Host genotype does not strongly influence the assemblage of microbial communities

In this study, we analyzed two cultivars of wheat, Apache recognized as more resistant to FHB than Caphorn. Caphorn is carrying the giberellic-acid insensitive allele *Rht-D1b* transferred from the Japanese cultivar “Norin 10,” conferring increasing yield but suspected to reduce resistance to FHB (Voss et al., 2008; Sip et al., 2009) whereas Apache is carrying the wild type allele *Rht-D1a* (Holzapfel et al., 2008; Voss et al., 2008). Globally, the correspondence analysis (Figure 2), based on non-singleton species, evidenced an overlap between the microbial communities isolated from each cultivar, Caphorn showing however a more diverse bacterial community (Table 1). Thus, we did not find a clear effect of the host genotype on the structure of microbial communities in wheat. However, given that plants can actively control the diversity of their microbial communities by recruiting beneficial microorganisms, especially from the soil (Rosenblueth and Martínez-Romero, 2004; Hartmann et al., 2009), and through the production of intrinsic regulatory molecules and secondary metabolites, each cultivar, because of its genetic make-up, is assumed to select its own microbial community. Actually, many studies have shown that host genotype may influence, along with prevailing environmental conditions, the composition of endophytic communities, for example in potato plants (Manter et al., 2010), common bean (De Oliveira Costa et al., 2012), or cotton seedlings (Adams and Kloepper, 2002), as well as the diversity of the rhizosphere microbiome in maize (Peiffer and Ley, 2013) or of the phyllosphere mycobiome in cereals (Sapkota et al., 2015). But Hardoim et al. (2011) have shown that different rice cultivars select specific microorganisms to shape their inner microbial communities, either in different or similar ways, depending on the cultivars, leading sometimes to close microbial communities between different cultivars. And sometimes, host genotype is less important in structuring microbial communities than others environmental or biological factors (Mason et al., 2015). Especially, it was shown that pathogen attack can have a greater impact in shaping endophytic communities than the plant genotype (Reiter et al., 2002). In the present study, both cultivars were colonized by species causing FHB (as described by Xu and Nicholson, 2009) and their distribution did not accounted for a higher resistance of Apache to the disease, despite the known difference of susceptibility of both cultivars toward FHB. But resistance to FHB is known to be complex and significantly affected by the environment (Rudd et al., 2001). We can assume that plants from both cultivars studied, exhibiting similar sanitary conditions at the time of the sampling, were able to recruit a close endophytic core microbiome. In view to the results it would be interesting to compare, within a given genotype, plants heavily and weakly infected, in order to better assess the effect of endophyte communities on FHB resistance.

### Microbial communities are strongly shaped by the organs from which they were isolated

Results show that microbial communities are significantly different between roots and aerial organs of wheat (Figure 4, Table 2), as expected given the extreme difference of the habitats where they are living, in terms of their degree of exposure (to air, sun, wind, rain, and related moisture and aeration conditions) and availability of nutrients (Andreote et al., 2014). Three species were found as indicators for roots (Table 2), *Bacillus megaterium, M. bolleyi*, and *G. graminis*, all of them being known as root-colonizers (Kirk and Deacon, 1987a; Kildea et al., 2008; Lenc et al., 2015). Besides indicator species, several typical soil fungi, root associated pathogens, and endophytes, were present but not abundant. Soil-borne fungi isolated are either saprophytes such as *Clonostachys rosea, Doratomyces microsporus, Mortierella alpina, T. flavus* (Domsch et al., 1980), or coprophilic species such as *P. glutinans* (Cain, 1962). Typical root-pathogens were present such as *Ophiosphaerella* sp. (Câmara et al., 2000), *Ilyonectria macrodidyma* (Chaverri et al., 2011), *R. solani* (Goll et al., 2014), and *Fusarium redolens* (Taheri et al., 2011). *Periconia macrospinosa* is a typical class 4 dark-septate endophyte (Rodriguez et al., 2009), usually isolated from roots of grasses (Mandyam et al., 2012). Indicator taxa for aerial organs (Table 2) included a majority of fungal pathogens (Table 1), that colonized extensively those organs, and, interestingly, the phylloplane fungus *Aureobasidium protae* and six bacterial species. *Aureobasidium* species produce abundant extracellular polysaccharides of high viscosity allowing strong adhesion on leaves (Gaur et al., 2010), making easier colonization of leaves tissues as endophytes. Although bacteria are abundantly found in the rhizosphere of wheat plants (McSpadden Gardener and Weller, 2001; Velázquez-Sepúlveda et al., 2012; Yin et al., 2013; Donn et al., 2015), many *Bacillus, Paenibacillus*, and *Pantoea* species (one species of each genus was indicator of aerial organs in this study) are also considered to be ubiquitous plant epiphytes (McSpadden Gardener, 2004; Brady et al., 2009). Two others indicator species, *E. persicina* and *E. aphidicola*, reported as pathogens of peas (Santos et al., 2009; Zhang and Nan, 2014) may have survived on previous crop debris and infected wheat seedlings.

### Microbial communities show temporal variations

Results show strong temporal variations of the global microbial communities in wheat (Figures 5, 6, Table 3). Species may be categorized in three groups according to their dynamics (Figure 6, Table 3) and designated as early species for those which were prevalent at heading and declining later on, persistent species, either increasing in density from heading to mealy ripe or showing a peak at flowering, and late species appearing at mealy ripe. Early species were dominated by *B. subtilis*, a species known to grow in soil and in the rhizosphere of many plants (Earl et al., 2008). They included soil-borne fungal pathogens, causing devastating diseases on a broad host range, such as *R. solani* (Lemańczyk, 2012), abundantly found in European agricultural soils (Goll et al., 2014)*, S. sclerotiorum* (Bolton et al., 2006), and *B. cinerea* (Dean et al., 2012) or considered as minor pathogens, such as *M. bolleyi*, commonly found on cereals roots (Kirk and Deacon, 1987a; Fernandez and Holzgang, 2009). *M. graminicola*, the causal agent of the *S. tritici* blotch, an important foliar disease on winter wheat in Europe (Miedaner et al., 2013), was mostly isolated at heading although its cycle extends until wheat maturity. This fungus has a long latent period, growing as a biotroph, terminating by a switch to necrotrophic growth (Goodwin et al., 2011). As we have collected plants with the fewest possible symptoms of diseases, we evicted the plants showing the necrotic lesions on leaves and twigs that develop later, after infected cells collapse. The very common wood-decaying fungus *H. rosae* (Telleria et al., 2010) was also abundantly isolated at heading but only from Apache. Among all these early species, only *R. solani, H. rosae, S. sclerotiorum*, and *Bacillus cereus* were found as indicators of heading by statistical analysis.

Among persistent species, three peaked at flowering and were indicators of this stage, *P. trivialis*, the black head mold *C. allii* and the fungal pathogen *D. exitialis* causing leaf spots. Others indicators of flowering were *A. protae, Fusarium tricinctum*, and *Stenotrophomonas africana*, but they were not abundant. Several species increased in density until maturity, the “black head moulds,” *Alternaria* and *Epicoccum*, which have serious implications for the quality of milling wheat (Zare, 2013) and *M. nivale*, one of the main causal agents of FHB (Xu and Nicholson, 2009). The two first were recognized as indicators of mealy ripe stage. The take-all fungus *G. graminis*, the most important root disease of wheat worldwide (Freeman and Ward, 2004), was present at flowering and mealy ripe but not abundant. Indeed, this disease is easily controlled by cultural practices, particularly by crop rotation, cultivating non-susceptible break crops (Freeman and Ward, 2004), such as peas in this study. Among late species, *F. graminearum*, a major pathogen involved in FHB is known to spread within the wheat head. Several bacteria appeared late, particularly on aerials organs, such as *P. vagans* and *P. agglomerans*, considered as non-pathogenic (Brady et al., 2009; Smits et al., 2010) and *E. aphidicola*, or on roots such as *B. megaterium*. All these late species were indicators of mealy ripe stage.

Altogether, these results show that temporal variations were mainly driven by the succession of pathogens. Previous studies indicated significant differences in bacterial populations over the seasons in roots and leaves of soybean and rice (Mano et al., 2007; Zhang et al., 2011). Likewise, temporal variations in fungal endophytes communities have already been observed in cotton (Ek-Ramos et al., 2013), and in several wild plants including trees and herbaceous grassland plants (Mishra et al., 2012; Wearn et al., 2012; Zimmerman and Vitousek, 2012).

### Interest of isolated species in plant protection

Among all species isolated as endophytes in this study, several are candidates for evaluation for use as biological control agents against wheat diseases, based on their known effects in the literature against wheat pathogens, either as endophytes or in interaction outside of plants. Indeed, *B. subtilis* was shown in the literature to inhibit *F. graminearum* and *F. culmorum*, two major species responsible for FHB (Palazzini et al., 2009; Khezri et al., 2011; Alimi et al., 2012; Zhao et al., 2014) and to reduce the incidence of diseased wheat leaves infected with *Puccinia striiformis* f. sp. *tritici* (Li et al., 2013). Likewise, *Bacillus amyloliquefaciens* and *B. cereus* are known to control FHB agents in controlled conditions (Alimi et al., 2012; Dunlap et al., 2013). Gromadzka et al. (2009) have studied the potential of *C. rosea* to control pathogenic *Fusarium* species on cereals and to decompose the mycotoxins produced. *C. rosea* was also shown to reduce *F. graminearum* and *F. culmorum* sporulation on wheat straw (Luongo et al., 2005). *Pseudomonas fluorescens* was shown to reduce mycotoxins contamination by FHB agents in greenhouses and field conditions (Amein et al., 2008; Khan and Doohan, 2009; Alimi et al., 2012). Fluorescent pseudomonads can also be suppressive to the take-all disease of wheat due to *G. graminis* (Weller and Cook, 1983). Among filamentous fungi, *M. bolleyi* was also shown to control *G. graminis* (Kirk and Deacon, 1987b). *B. megaterium* can decrease *S. tritici* blotch up to 80% under environmental controlled conditions (Kildea et al., 2008). The yeast *R. kratochvilovae*, was shown to reduce the disease caused by *B. graminis* f. sp. *tritici* and increase grain yield of durum wheat (De Curtis et al., 2012). *Chaetomium* spp. were found to reduce the number and area of pustules caused by *Puccinia triticina* (Dingle and McGee, 2003) and to inhibit *Pyrenophora tritici in vitro* (Istifadah et al., 2006).

Some species are known as biological control agents against others plant diseases. *E. nigrum* is known as biological control agent against fungal pathogens such as *B. cinerea* (Alcock et al., 2015) or *Monilinia laxa* on fruits surface (Larena et al., 2005). *Bacillus pumilus* is also known to inhibit several plant pathogens such as *Fusarium solani* on tomato (Ajilogba et al., 2013) or fungal pathogens responsible for poplar canker (Ren et al., 2013). *P. vagans* and *P. agglomerans* have demonstrated strong beneficial activities as biological control of bacterial diseases (Johnson and Stockwell, 1998; Braun-Kiewnick et al., 2000). *P. trivialis* produces numerous volatile compounds able to inhibit the growth of pathogens such as *R. solani* on lettuce (Scherwinski et al., 2008). *Athelia bombacina* has been reported as an antagonist of apple scab caused by *Venturia inaequalis* (Fiaccadori and Cesari, 1998). *Oxyporus latemarginatus* was shown to produce an antifungal volatile compound controlling *B. cinerea* and *R. solani* on apple and moth orchid respectively (Lee et al., 2009). Kakvan et al. (2013) demonstrated that *T. flavus* might be a potential biological control agent against *R. solani*-induced sugar beet damping-off disease. Finally, culture filtrates of *T. versicolor* were shown to reduce the production of toxin by *Aspergillus flavus* on maize (Scarpari et al., 2014). Therefore these species, unknown so far for the control of wheat diseases, may also have a potential in plant protection.

It is of interest to note that among the species previously reported as biological control agents in the literature and isolated as non-singleton in this study, some have been isolated only from aerial organs of plants (e.g., *B. amyloliquefaciens, C. globosum, A. bombacina, E. nigrum, O. latemarginatus*, Figure 3) or only from roots (e.g., *B. cereus, B. megaterium, B. pumilus, M. bolleyi, T. flavus*, Figure 3), revealing the importance to sample several organs in plants when conducing a survey on endophytes, in order to increase the collection of microorganisms with potential beneficial applications. Likewise, some species have been isolated at only one stage of plant development, for example *B. cereus, C. globosum, A. bombacina, O. latemarginatus*, and *B. pumilus* have been recovered only at heading (Figure 3) whereas *B. amyloliquefaciens, P. agglomerans, P. vagans*, and *T. flavus* were recovered only at mealy ripe (Figure 3), pointing out the interest of sampling plants at different stages of maturity in order to maximize the number of species isolated.

Among all isolated species, those unknown in plant protection but not reported as plant pathogens represent a reservoir of potential new biological control agents and might therefore be worth investigating against wheat diseases. Species isolated as non-singleton, such as *C. gallica, G. idaeicola, D. cejpii, P. glutinans*, or *P. lepideus* could be particularly interesting, given their ability to co-occur with plant pathogens.

## Conclusion

This study reveals that an important diversity of fungi and bacteria is able to live as endogenous microbes in wheat, in spite of the clear dominance of fungal pathogens of wheat, and for some species this is the first report of being isolated as wheat endophytes. We analyzed two cultivars, expecting an effect of host genotype on microbial communities, particularly on FHB agents. But we did not detect any real effect of the host genotype on microbial communities. Microbial communities have shown however strong spatial and temporal variations. They were highly structured by the host organ from which they were isolated, aerial parts vs. roots. As expected, roots were mostly colonized by soil-inhabiting bacteria and fungi, whereas aerials organs were particularly colonized by fungal pathogens, class 3 fungal endophytes and several bacterial species known as epiphytes. Temporal variations were mainly driven by the succession of pathogens. This better characterization of microbial diversity and distribution patterns in wheat plants will help to develop microorganisms that could be exploited in integrated pest management. Sampling different parts of plants and at different times of plant maturity substantially increased the richness of microorganisms recovered. The clear advantage of this study is the establishment of a collection of cultivable fungal and bacterial endophytes that can now be evaluated for their ability to have a protective effect against wheat pathogens. Should any of these microorganisms be further developed as biological control agents, our analysis of variation in microbial communities assemblages suggest that their establishment in wheat plants will not be restricted to specific cultivars.

## Author contributions

Conceived and designed the experiments: CP and JD. Performed the experiments: MC and SL. Analyzed the data: MC and JD. Contributed reagents/materials/analysis tools: MC and SL. Wrote the paper: MC, FB, and JD.

## Funding

This work was supported by the public funding agency Bpifrance (grant OSIRIS) and the Ets J. Soufflet.

### Conflict of interest statement

The authors declare that the research was conducted in the absence of any commercial or financial relationships that could be construed as a potential conflict of interest.
