# Supplementary material for: Spatial and Temporal Variation of Cultivable Communities of Co-occurring Endophytes and Pathogens in Wheat
Source: Front Microbiol. 2016 Mar 31;7:403. doi: 10.3389/fmicb.2016.00403 (PMC4814462; doi:10.3389/fmicb.2016.00403)
Supplement: Supplementary file 1 [file Table1.DOCX]

**S1 Table.** Diversity indices measuring species richness in each of the 24 wheat plants sampled from the two wheat cultivars Caphorn and Apache, at three stages of development. Frequency of singletons in each plant is also reported.

| Caphorn | | | | | | | | | | | | | | | | | | | | | | | | |
| --- | --- | --- | --- | --- | --- | --- | --- | --- | --- | --- | --- | --- | --- | --- | --- | --- | --- | --- | --- | --- | --- | --- | --- | --- |
| Stages | Heading | | | | | | | | Flowering | | | | | | | | Mealy Ripe | | | | | | | |
| Wheat plants | P1 | | P2 | | P3 | | P4 | | P5 | | P6 | | P7 | | P8 | | P9 | | P10 | | P11 | | P12 | |
| Shannon index | 3.31 | | 3.36 | | 3.37 | | 2.56 | | 2.36 | | 2.12 | | 2.65 | | 2.91 | | 3.26 | | 3.66 | | 3.68 | | 3.40 | |
| Shannon index in aerial organs (A) or roots (R) | A | R | A | R | A | R | A | R | A | R | A | R | A | R | A | R | A | R | A | R | A | R | A | R |
|  | 2.96 | 1.30 | 3.08 | 2.32 | 2.32 | 2.69 | 1.37 | 2.06 | 2.45 | 1.15 | 1.89 | 1.55 | 2.47 | 1.96 | 2.72 | 1.90 | 2.97 | 1.79 | 3.16 | 2.41 | 3.11 | 2.16 | 3.26 | 0 |
| Pielou index | 0.92 | | 0.91 | | 0.97 | | 0.81 | | 0.79 | | 0.67 | | 0.74 | | 0.84 | | 0.91 | | 0.91 | | 0.92 | | 0.95 | |
| Frequency of singletons (%) | 9.10 | | 7.70 | | 9.10 | | 0 | | 11.1 | | 11.1 | | 0 | | 8.30 | | 0 | | 12.5 | | 6.30 | | 0 | |
| Apache | | | | | | | | | | | | | | | | | | | | | | | | |
| Stages | Heading | | | | | | | | Flowering | | | | | | | | Mealy Ripe | | | | | | | |
| Wheat plants | P13 | | P14 | | P15 | | P16 | | P17 | | P18 | | P19 | | P20 | | P21 | | P22 | | P23 | | P24 | |
| Shannon index | 3.33 | | 3.90 | | 3.31 | | 3.57 | | 2.97 | | 2.45 | | 2.76 | | 2.90 | | 3.58 | | 3.21 | | 3.50 | | 3.59 | |
| Shannon index in aerial organs (A) or roots (R) | A | R | A | R | A | R | A | R | A | R | A | R | A | R | A | R | A | R | A | R | A | R | A | R |
|  | 3.35 | 0.65 | 3.62 | 2.11 | 3 | 1.75 | 3.39 | 2.06 | 2.68 | 1 | 2.45 | 0 | 2.32 | 2 | 2.59 | 1.37 | 3.37 | 1.25 | 2.82 | 1.84 | 3.05 | 1.66 | 3.13 | 2.13 |
| Pielou index | 0.90 | | 0.92 | | 0.89 | | 0.89 | | 0.89 | | 0.77 | | 0.80 | | 0.84 | | 0.94 | | 0.90 | | 0.92 | | 0.94 | |
| Frequency of singletons (%) | 14.3 | | 15.8 | | 15.4 | | 14.3 | | 0 | | 0 | | 0 | | 0 | | 7.10 | | 0 | | 0 | | 0 | |
