# Supplementary material for: Spatial and Temporal Variation of Cultivable Communities of Co-occurring Endophytes and Pathogens in Wheat
Source: Front Microbiol. 2016 Mar 31;7:403. doi: 10.3389/fmicb.2016.00403 (PMC4814462; doi:10.3389/fmicb.2016.00403)
Supplement: Supplementary file 2 [file Table2.DOCX]

| Species | Sieber *et al.* 1988 | Crous *et al.* 1995 | Larran *et al.* 2007 | Vujanovic *et al*. 2012 | Lenc *et al.* 2015 |
| --- | --- | --- | --- | --- | --- |
| *Alternaria spp* | + | + | + | + | - |
| *Aureobasidium spp* | - | - | - | - | + |
| *Clonostachys rosea* | - | - | - | - | + |
| *Chaetomium globosum* | - | + | + | + | + |
| *Cladosporium herbarum complex* | + | + | + | + | + |
| *Didymella exitialis* | + | + | - | - | - |
| *Epicoccum nigrum* | + | + | + | + | + |
| *Fusarium tricinctum* | - | - | - | + | + |
| *Gaeumannomyces graminis* | - | - | - | - | *+* |
| *Fusarium graminearum* | + | - | + | - | + |
| *Ilyonectria macrodidyma* | - | + | - | - | + |
| *Microdochium bolleyi* | + | + | - | + | + |
| *Microdochium nivale* | + | - | - | - | - |
| *Mortierella spp* | - | - | - | + | + |
| *Mycosphaerella / Septoria spp* | + | + | + | - | - |
| *Ophiosphaerella sp.* | - | - | - | + | - |
| *Periconia macrospinosa* | - | + | - | + | + |
| *Phaeosphaeria/Parastagonospora* | - | - | - | + | - |
| *Phoma spp* | - | + | + | + | + |
| *Pyrenophora tritici-repentis* | - | + | - | + | - |
| *Rhizoctonia solani* | + | - | - | - | + |
| *Sarocladium/Acremonium /Cephalosporium spp* | - | + | + | + | + |

**S2 Table.** Fungal species common to previous studies on wheat fungal colonizers.
