# Supplementary figures and images for: Spatial and Temporal Variation of Cultivable Communities of Co-occurring Endophytes and Pathogens in Wheat
Source: Front Microbiol. 2016 Mar 31;7:403. doi: 10.3389/fmicb.2016.00403 (PMC4814462; doi:10.3389/fmicb.2016.00403)

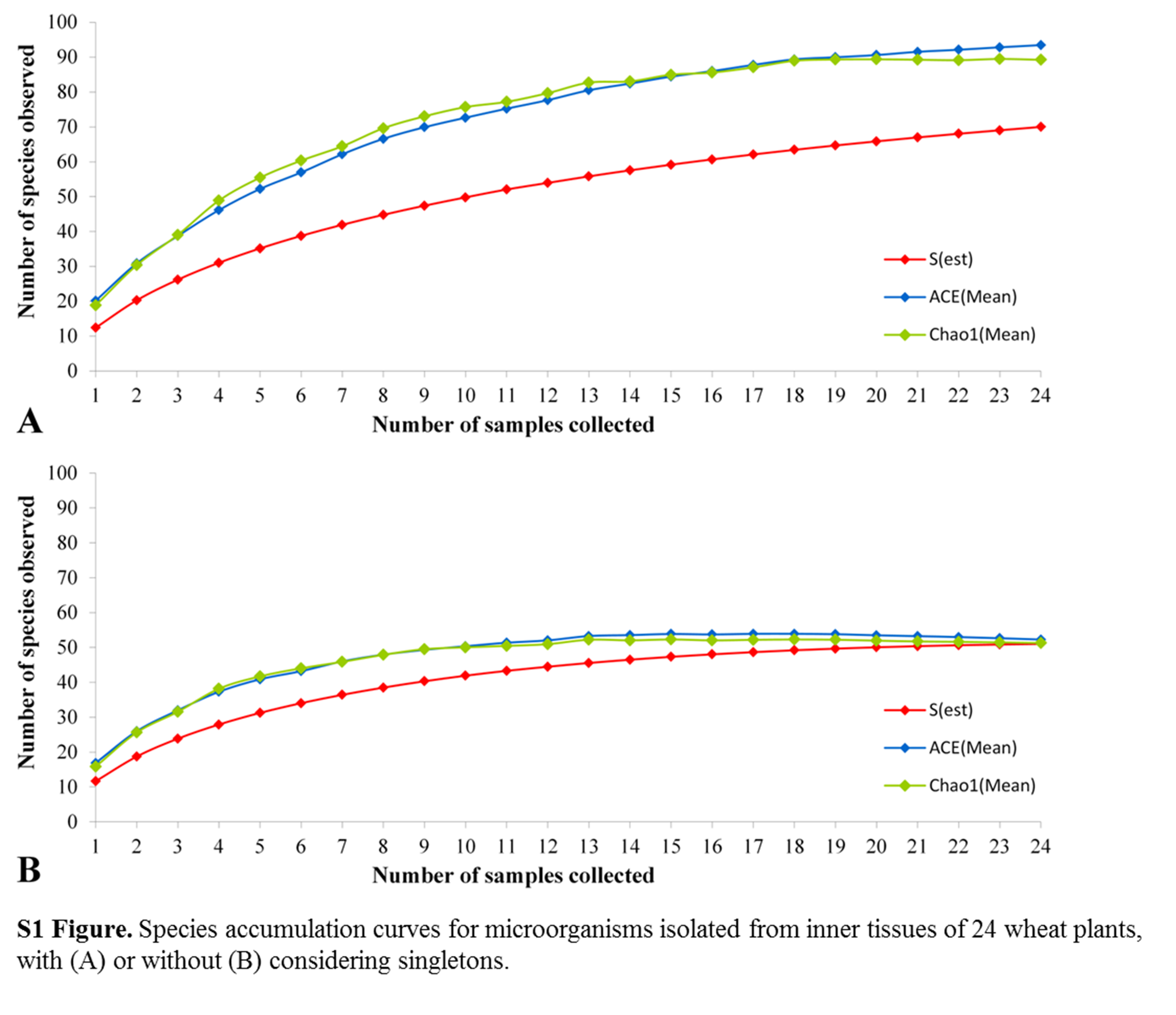

Supplement: Supplementary file 3 [file Image1.TIF]
